# Systematic Nucleotide Exchange Analysis of ESTs From the Human Cancer Genome Project Report: Origins of 347 Unknown ESTs Indicate Putative Transcription of Non-Coding Genomic Regions

**DOI:** 10.3389/fgene.2020.00042

**Published:** 2020-02-11

**Authors:** Ganesh Warthi, Pierre-Edouard Fournier, Hervé Seligmann

**Affiliations:** ^1^ Aix Marseille Univ, IRD, APHM, SSA, VITROME, IHU-Méditerranée Infection, Marseille, France; ^2^ IHU-Méditerranée Infection, Marseille, France; ^3^ The National Natural History Collections, The Hebrew University of Jerusalem, Jerusalem, Israel; ^4^ Université Grenoble Alpes, Faculty of Medicine, Laboratory AGEIS EA 7407, Team Tools for e-Gnosis Medical & Labcom CNRS/UGA/OrangeLabs Telecoms4Health, La Tronche, France

**Keywords:** swinger RNAs, systematic nucleotide exchanges, transcriptome, cDNA, cancer

## Abstract

Expressed sequence tags (ESTs) provide an imprint of cellular RNA diversity irrespectively of sequence homology with template genomes. NCBI databases include many unknown RNAs from various normal and cancer cells. These are usually ignored assuming sequencing artefacts or contamination due to their lack of sequence homology with template DNA. Here, we report genomic origins of 347 ESTs previously assumed artefacts/unknown, from the FAPESP/LICR Human Cancer Genome Project. EST template detection uses systematic nucleotide exchange analyses called swinger transformations. Systematic nucleotide exchanges replace systematically particular nucleotides with different nucleotides. Among 347 unknown ESTs, 51 ESTs match mitogenome transcription, 17 and 2 ESTs are from nuclear chromosome non-coding regions, and uncharacterized nuclear genes. Identified ESTs mapped on 205 protein-coding genes, 10 genes had swinger RNAs in several biosamples. Whole cell transcriptome searches for 17 ESTs mapping on non-coding regions confirmed their transcription. The 10 swinger-transcribed genes identified more than once associate with cancer induction and progression, suggesting swinger transformation occurs mainly in highly transcribed genes. Swinger transformation is a unique method to identify noncanonical RNAs obtained from NGS, which identifies putative ncRNA transcribed regions. Results suggest that swinger transcription occurs in highly active genes in normal and genetically unstable cancer cells.

## Introduction

**Figure 1:**
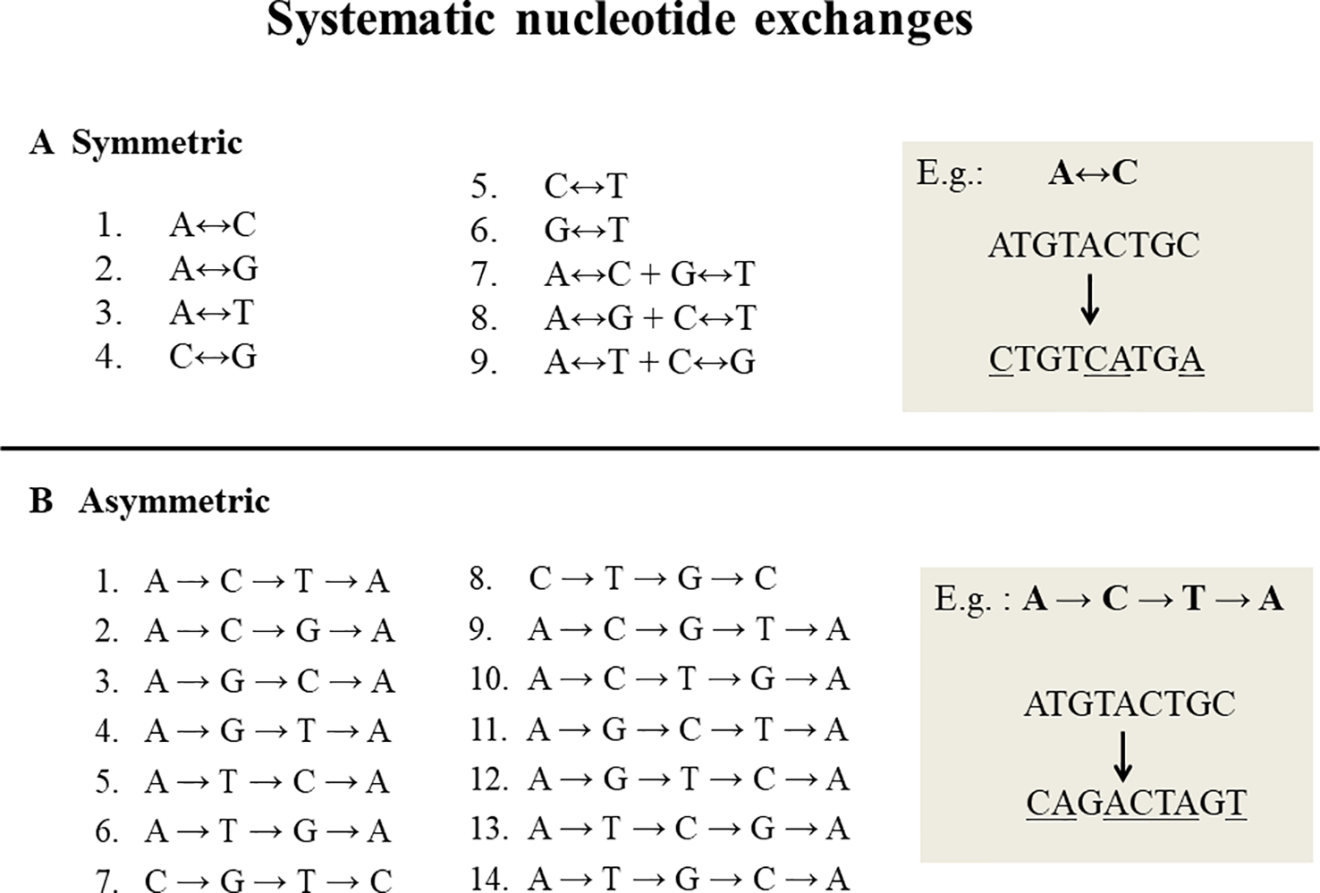
Twenty three systematic nucleotide exchanges. **(A)** 9 Symmetric exchanges in which one nucleotide is exchanged by another during DNA replication or transcription, e.g. A ↔ C, where all As are replaced by Cs and all Cs by As. **(B)** 14 Asymmetric exchanges that are directional in nature, e.g. A→C→T→A: all As are replaced by Cs, Cs by Ts and Ts by As.

Systematic nucleotide exchanges, also called swinger transformations, systematically exchange specific nucleotides with other specific nucleotides during DNA replication and/or RNA transcription. DNA or RNA molecules corresponding to systematic nucleotide changes are called swinger DNAs or swinger RNAs. Only 23 systematic nucleotide exchanges (**Figure 1**) are possible with four nucleotide bases (A, T, C and G), i.e. nine symmetric (X ↔ Y, e.g. A ↔ G) (Seligmann, 2013a; Seligmann, 2013b) and 14 asymmetric (X → Y → Z → X, e.g. A → G → T → A) (Seligmann, 2013b; Seligmann, 2013c). For example, in symmetric exchange A ↔ G, all As are replaced by Gs and all Gs by As. The 14 asymmetric exchanges are directional, e.g. A → G → T → A: all As are replaced by Gs, Gs by Ts and Ts by As. It is unclear whether these systematic exchanges occur during DNA/RNA polymerizations or result from posttranscriptional editions. Their relatively long lengths (> 100 nucleotides) favors the former. Previous correlation analyses (Seligmann, 2013b; Seligmann, 2013c; Michel and Seligmann, 2014) show that the lengths and abundances of swinger RNAs are approximately proportional to rates calculated on the basis of corresponding single nucleotide misinsertions by the human mitochondrial gamma DNA polymerase (from Lee and Johnson, 2006). This suggests that swinger RNAs result from polymerizations where the polymerase is stabilized in the usually transient, unstable state that causes regular single nucleotide misinsertions.

Systematic nucleotide exchange analyses identify genomic origins of unknown DNA/RNA sequences. Several types of RNAs with no sequence homology to template genomes exist. This includes RNA-DNA differences (Blank et al., 1986; Ninio, 1991; Li et al., 2011; Bahn et al., 2012; Strathern et al., 2012; Bar-Yaacov et al., 2013; Knippa and Peterson, 2013; Zhou et al., 2013; Wang et al., 2016), post-transcriptional editing (Bass, 2002; Schaub and Keller, 2002; Chen and Bundschuh, 2012; Park et al., 2012; Wang IX et al, 2014a; Lee et al., 2015), post-transcriptional hyper editing (Porath et al., 2014) and polymerase template switching (Lee et al., 2007; Löytynoja and Goldman, 2017). Transcript fusion explains some noncanonical RNAs (Kumar et al., 2016; López-Nieva et al., 2019; Singh et al., 2019). Some rare DNAs and RNAs seerm to result from spontaneous, template-free polymerization (Béguin et al., 2015; Seligmann and Raoult, 2018). Due to the systematic nature of swinger transformations, identified, previously unknown sequence reads overcome the widely argued possibility of by-chance alignment with randomly amplified sequencing artefacts since randomness does not produce systematic exchanges.

Mitogenomic human swinger RNA reads produced by Illumina confirmed corresponding EST data (Seligmann, 2016b). The swinger transcriptome of the amoeban-hosted Mimivirus was also confirmed by two different sequencing techniques (SOLID and 454, Seligmann and Raoult, 2018). Detected peptides matching translation of the swinger-transformed mitogenome tend to map on detected swinger RNAs (Seligmann, 2016a; Seligmann, 2016b; Seligmann, 2016c; Seligmann, 2017a). These findings were further confirmed in purified mitochondrial transcriptomes (Warthi and Seligmann, 2018), rejecting the possibility of cytoplasmic contamination. Chimeric mitochondrial swinger RNAs also exist, partly following swinger polymerization and partly regular polymerization, with abrupt switches between these parts (Seligmann, 2015a; Seligmann, 2015b). This observation is paralleled by chimeric peptides, corresponding to translation of adjacent regular and swinger RNA (Seligmann, 2016d). Swinger RNA coverage associates with secondary structure formation (Seligmann, 2016e). Swinger DNA also occurs (Seligmann, 2014a; Seligmann, 2014b). Molecular functions and associations of swinger polymerizations with healthy or unhealthy cells remain unknown. Recently, swinger RNAs with A ↔ T + C ↔ G transformation were identified in HIV mediated Non-Hodgkin's Lymphoma (Warthi et al., 2019). The identified RNAs with A ↔ T + C ↔ G transformation are similar to the transcriptional product by polymerase template switching, known in retroviruses (Kandel and Nudler, 2002) and the human genome (Löytynoja and Goldman, 2017).

The Encyclopedia of DNA Elements (ENCODE) project concluded that a human genome includes approximately 20,000 protein-coding genes and predicts that 80% of non-coding regions regulate gene expression (Dunham et al., 2012). However, the transcriptomes generated by current RNA sequencing technologies do not cover the complete human genome, because analyses of sequencing reads assume only canonical transcription. Analyses assuming systematic nucleotide exchanges could identify non-canonical RNAs and their genomic origin.

Expressed sequence tags (ESTs) are short DNA sequences (100–600 bp) generated from the sequencing of cDNA libraries. The ESTs represent RNAs derived from a particular cell irrespective of sequence similarity or dissimilarity to its template genome, unlike transcriptomes, in which sequencing reads with sequence similarity are considered as true reads while ignoring non-homologous reads. This underlines a major limitation in identifying and studying non-canonical RNAs and their association with various genetic diseases like cancers. Therefore, here we apply swinger transformations to identify unknown expressed sequence tags (ESTs) occurring in the FAPESP/LICR Human Cancer Genome Project (Neto et al., 2000). We expect to identify unknown ESTs reported in cancer cells, identify and report their genomic origins and confirm that swinger-transformed RNAs occur beyond mitochondria.

## Methods

### Identification of Unknown Ests

The FAPESP/LICR Human Cancer Genome Project corresponds to 891,011 published (Neto et al., 2000) and 55,248 unpublished ESTs in the NCBI database. These ESTs were blasted with the “Human genomic + transcript” database using the “highly similar sequence” algorithm (megablast) (Zhang et al, 2000; Morgulis et al., 2008). A total of 149,500 ESTs with no sequence similarity with the human genome were further blasted across all sequences in the NCBI database to exclude ESTs corresponding to potential contaminations.

### Swinger Transformation of Unknown Ests

The unknown 149,500 ESTs were then swinger-transformed according to the 23 bijective transformations (**Figure 1**) (Michel and Seligmann, 2014). These 149,500 unknown ESTs were blasted again with the “Human genomic + transcript” database using megablast, in order to detect their genomic template.

## Results and Discussion

**Figure 2:**
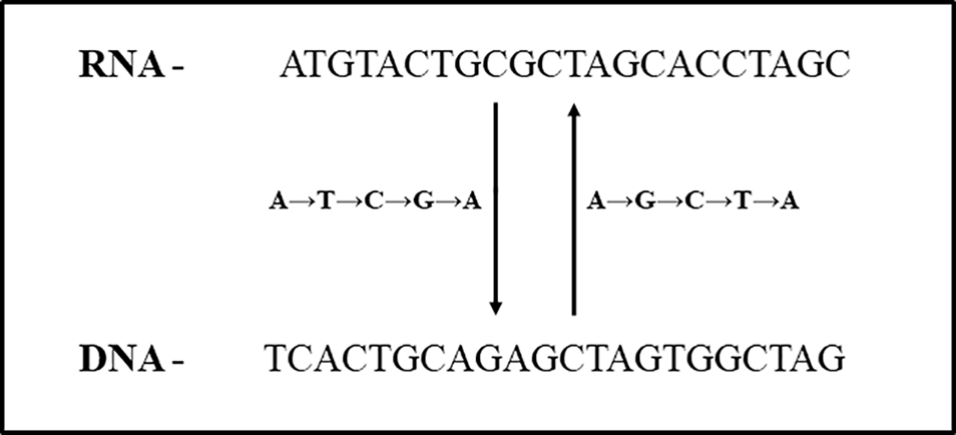
Relationship between A → T → C → G → A and A → G → C → T → A asymmetric swinger transformation.

In total, 347 ESTs (0.23%) were identified using swinger transformation analyses (**Tables 1**–**3**). This rate is 4× lower than that estimated for human mitochondria (about 100/10000 ESTs, 1% (data from Seligmann, 2012; Seligmann, 2013b; Seligmann, 2013c). Swinger-transformed sequences of these identified 347 ESTs are in **Supplementary Table 1**. The remaining 99.77% of unidentified ESTs could be sequencing artefacts. These could also be ESTs with non-systematic post-transcriptional hyper editing (Porath et al., 2014) or resulting from systematic nucleotide deletion transcription (Warthi and Seligmann, 2019). Among 347 ESTs, 223 ESTs were symmetrically transformed swinger RNAs (219 ESTs were A ↔ T transformed, two ESTs were A ↔ C transformed, and one of each was C ↔ T and G ↔ T transformed). The average length of alignment of A ↔ T, A ↔ C, C ↔ T and G ↔ T transformed ESTs was 325 bp, 225 bp, 196 bp, 152 bp with an average percentage identity of 96.95, 90.52, 96.42, and 89.47% respectively. Similarly, A → T → C → G → A transformed asymmetric swinger transformations account for 124 ESTs with 96.95% average percentage identity and 308 bp average alignment length. The largest aligned swinger RNA was 685 bp with 41 bp as the smallest. Unlike symmetrical transformations, ESTs identified as A → T → C → G → A transformed have to be transcribed assuming A → G → C → T → A from the template DNA region (**Figure 2**). From here on, these identified ESTs are considered swinger RNAs. Abundances of swinger RNA classes are proportional to their mean length (r = 0.96, two tailed P = 0.00945). Assuming that different swinger classes result from different polymerase states, the positive correlation indicates that the same factor that promotes switching to a given mode of swinger transcription also favors the stability of this mode. The opposite, indicated by a negative association, would mean that frequent types of switches are unstable. We suggest that systematic nucleotide transformations happen during transcription (swinger transcription). This might result from polymerase enzyme fatigue. In carcinogenesis, during malignant transformation, cancer cells produce mutated RNAs and proteins because of genetic instability (Ciocca and Calderwood, 2005; Ruckova et al., 2012). Therefore, swinger RNAs are probably mainly non- or dys-functional, and produce non- and dysfunctional proteins with probable carcinogenic effects.

**Table 1:** List of swinger transformed ESTs identified using BLAST. The list includes ESTs identified using swinger transformations and mapped on genes identified only in one biosamples.

EST Acc No.	Biosample	Cancer tissue	EST Length	Swinger transformation	Aligned sequence AI	Chromosome	Gene	Aligned Seq. length	%ID	e-value	5'	3'
AI902333.1	SAMN00156544	Breast	173	A ↔ C	NM_001278462.1	12	MDM2 proto-oncogene (MDM2)	100	97.000	2.37e−39	65	163
AI903883.1	SAMN00156581	Breast	193	G ↔ T	NM_080386.3	2	tubulin alpha 3d (TUBA3D)	147	93.197	4.39e−57	1,095	950
AI939933.1	SAMN00156800	Colon	283	A ↔ T	NM_001185092.1	1	nitrilase 1 (NIT1)	168	94.643	6.67e−67	402	238
AI939934.1*	SAMN00156800	Colon	273	A ↔ T	NM_001037333.2	5	cytoplasmic FMR1 interacting protein 2 (CYFIP2)	200	96.500	1.80e−87	738	540
AI939935.1*	SAMN00156800	Colon	156	A ↔ T	NM_001037333.2	5	cytoplasmic FMR1 interacting protein 2 (CYFIP2)	142	95.775	1.45e−58	733	592
AI939936.1*	SAMN00156800	Colon	303	A ↔ T	NM_001037333.2	5	cytoplasmic FMR1 interacting protein 2 (CYFIP2)	202	98.515	1.78e−97	741	540
AI939937.1	SAMN00156800	Colon	253	A ↔ T	NM_001008491.2	2	septin 2 (SEPT2)	234	98.291	6.26e−112	2,935	2,703
AI939938.1*	SAMN00156800	Colon	266	A ↔ T	NM_001037333.2	5	cytoplasmic FMR1 interacting protein 2 (CYFIP2)	202	94.059	8.44e−81	541	741
AI939939.1	SAMN00156800	Colon	377	A ↔ T	NW_019805496.1	11	NADH:ubiquinone oxidoreductase core subunit S3 (NDUFS3)	246	98.374	1.34e−118	10,294	10,049
AI939940.1	SAMN00156800	Colon	361	A ↔ T	NM_001008491.2	2	septin 2 (SEPT2)	299	94.314	2.85e−125	2,905	2,608
AI939941.1	SAMN00156801	Colon	303	A ↔ T	NM_001267774.1	17	intraflagellar transport 20 (IFT20)	220	98.182	1.05e−104	609	390
AI939942.1	SAMN00156801	Colon	182	A ↔ T	NC_000008.11	8	vir like m6A methyltransferase associated (VIRMA)	77	97.403	5.45e−28	94,525,717	94,525,793
AI939943.1	SAMN00156801	Colon	312	A ↔ T	NM_001282538.1	1	G protein subunit beta 1 (GNB1)	301	96.013	1.01e−134	1,212	1,512
AI939947.1*	SAMN00156461	Colon	210	A ↔ T	NM_001009570.2	2	chaperonin containing TCP1 subunit 7 (CCT7)	182	95.604	5.08e−78	1,127	946
AI939949.1*	SAMN00156461	Colon	622	A ↔ T	NM_001009570.2	2	chaperonin containing TCP1 subunit 7 (CCT7)	572	94.231	0.0	576	1,143
AI939950.1*	SAMN00156461	Colon	512	A ↔ T	NM_001009570.2	2	chaperonin containing TCP1 subunit 7 (CCT7)	484	98.347	0.0	1,183	702
AI939951.1*	SAMN00156802	Colon	317	A ↔ T	NR_037849.1	2	WW domain binding protein 1 (WBP1) INO80B-WBP1 readthrough (NMD candidate) (INO80B-WBP1), long non-coding RNA	310	94.839	4.70e−133	1,103	796
AI939953.1	SAMN00156802	Colon	609	A ↔ T	NM_001134671.2	8	derlin 1 (DERL1)	608	97.862	0.0	2,407	1,802
AI939959.1*	SAMN00156802	Colon	218	A ↔ T	NR_037849.1	2	WW domain binding protein 1 (WBP1) INO80B-WBP1 readthrough (NMD candidate) (INO80B-WBP1), long non-coding RNA	219	97.717	6.34e−102	1,043	826
AI939961.1*	SAMN00156802	Colon	231	A ↔ T	NM_033017.3	7	tripartite motif containing 4 (TRIM4)	204	95.588	2.33e−86	2,432	2,633
AI939962.1*	SAMN00156802	Colon	212	A ↔ T	NM_001300821.2	12	eukaryotic translation initiation factor 4B (EIF4B)	160	96.250	6.67e−67	2,434	2,276
AI939963.1*	SAMN00156802	Colon	340	A ↔ T	NM_033017.3	7	tripartite motif containing 4 (TRIM4)	291	95.189	2.85e−125	2,629	2,344
AI939965.1	SAMN00156802	Colon	214	A ↔ T	NR_038854.1	12	negative regulator of antiviral response (non-protein coding) (NRAV), long non-coding RNA	220	94.545	1.39e−88	632	848
AI939966.1*	SAMN00156802	Colon	366	A ↔ T	NR_037849.1	2	WW domain binding protein 1 (WBP1) INO80B-WBP1 readthrough (NMD candidate) (INO80B-WBP1), long non-coding RNA	317	94.637	2.81e−135	1,717	2,032
AI939968.1	SAMN00156802	Colon	270	A ↔ T	NC_000003.12	3	CUB domain containing protein 1 (CDCP1)	270	98.889	1.31e−133	45,123,674	45,123,943
AI939969.1*	SAMN00156802	Colon	143	A ↔ T	NM_001300821.2	12	eukaryotic translation initiation factor 4B (EIF4B)	123	98.374	6.63e−54	2,313	2,434
AI939970.1*	SAMN00156802	Colon	255	A ↔ T	NM_033017.3	7	tripartite motif containing 4 (TRIM4)	231	94.805	1.04e−96	2,406	2,633
AI939972.1	SAMN00156802	Colon	168	A ↔ T	NC_000017.11	17	target of myb1 like 2 membrane trafficking protein (TOM1L2)	166	96.386	1.39e−70	17,849,628	17,849,465
AI939973.1	SAMN00156802	Colon	337	A ↔ T	NR_037616.1	6	thioredoxin domain containing 5 (TXNDC5) and BLOC1S5-TXNDC5 readthrough (NMD candidate) (BLOC1S5-TXNDC5), long non-coding RNA	340	97.059	4.42e−160	1,880	1,542
AI939976.1*	SAMN00156802	Colon	200	A ↔ T	NR_037849.1	2	WW domain binding protein 1 (WBP1) INO80B-WBP1 readthrough (NMD candidate) (INO80B-WBP1), long non-coding RNA	200	100.000	1.34e−100	1,785	1,984
AI939980.1	SAMN00156802	Colon	155	A ↔ T	NM_144570.2	16	Jupiter microtubule associated homolog 2 (JPT2)	156	94.872	3.04e−62	2,949	2,794
AI939981.1*	SAMN00156802	Colon	229	A ↔ T	NR_037849.1	2	WW domain binding protein 1 (WBP1) INO80B-WBP1 readthrough (NMD candidate) (INO80B-WBP1), long non-coding RNA	235	96.170	2.22e−103	1,996	1,762
AI939988.1*	SAMN00156802	Colon	290	A ↔ T	NR_037849.1	2	WW domain binding protein 1 (WBP1) INO80B-WBP1 read through (NMD candidate) (INO80B-WBP1), long non-coding RNA	280	96.786	7.71e−128	2,021	1,744
AI940584.1*	SAMN00156483	Head_neck	583	A ↔ T	NM_001166285.1	2	chaperonin containing TCP1 subunit 7 (CCT7)	568	93.838	0.0	1,890	1,326
AW062762.1	SAMN00156993	Colon	433	A ↔ T	NM_001354666.1	3	peroxisome proliferator activated receptor gamma (PPARG), transcript	409	98.778	0.0	1,137	1,545
AW062764.1	SAMN00156993	Colon	610	A ↔ T	NC_000002.12	2	protein kinase C epsilon (PRKCE)	491	99.389	0.0	45,959,855	45,959,366
AW062765.1	SAMN00156993	Colon	360	A ↔ T	NC_000009.12	9	sorting nexin family member 30 (SNX30)	341	99.120	2.01e−173	112,811,006	112,811,345
AW062769.1	SAMN00156993	Colon	595	A ↔ T	NM_001198773.2	1	phosphatidylinositol 4-kinase beta (PI4KB)	466	98.712	0.0	3,151	3,615
AW062770.1	SAMN00156993	Colon	589	A ↔ T	NC_000016.10	16	Rho GTPase activating protein 17 (ARHGAP17)	586	99.147	0.0	24,921,862	24,922,445
AW062771.1	SAMN00156993	Colon	643	A ↔ T	NM_006386.4	22	DEAD-box helicase 17 (DDX17)	605	96.694	0.0	1,878	2,480
AW062772.1	SAMN00156993	Colon	540	A ↔ T	NM_000381.3	X	midline 1 (MID1)	519	99.037	0.0	5,758	5,240
AW062773.1	SAMN00156993	Colon	378	A ↔ T	NM_002795.3	17	proteasome subunit beta 3 (PSMB3)	368	98.913	0.0	384	751
AW062775.1	SAMN00156993	Colon	650	A ↔ T	NM_000358.2	5	transforming growth factor beta induced (TGFBI)	325	97.846	2.05e−158	1,166	1,489
AW062776.1	SAMN00156993	Colon	722	A ↔ T	XM_017008304.2	4	PREDICTED: Homo sapiens RNA binding motif protein 47 (RBM47)	357	97.479	9.36e−172	1,924	2,280
AW062777.1	SAMN00156993	Colon	449	A ↔ T	NM_001345950.1	10	tripartite motif containing 8 (TRIM8)	110	92.727	4.07e−36	1,009	901
AW062778.1	SAMN00156993	Colon	491	A ↔ T	NC_000010.11	10	C-terminal binding protein 2 (CTBP2)	474	98.734	0.0	125,121,894	125,121,421
AW062779.1	SAMN00156993	Colon	252	A ↔ T	NM_001136153.1	6	activating transcription factor 6 beta (ATF6B)	234	98.718	6.09e−114	1,207	1,440
AW062782.1	SAMN00156993	Colon	558	A ↔ T	NC_000008.11	8	ArfGAP with SH3 domain, ankyrin repeat and PH domain 1 (ASAP1)	356	95.787	3.41e−161	130,131,998	130,132,351
AW062784.1	SAMN00156993	Colon	542	A ↔ T	NM_015397.3	9	DDB1 and CUL4 associated factor 12 (DCAF12)	326	97.546	4.45e−155	2,795	3,116
AW062787.1	SAMN00156993	Colon	647	A ↔ T	NC_000012.12	12	signal transducer and activator of transcription 2	608	91.776	0.0	56,349,156	56,348,551
AW062788.1	SAMN00156993	Colon	458	A ↔ T	NC_000021.9	21	VPS26 endosomal protein sorting factor C (VPS26C)	293	97.611	4.54e−140	37,236,577	37,236,868
AW062789.1	SAMN00156993	Colon	685	A ↔ T	NC_000017.11	17	Myosin XVIIIA (MYO18A)	406	98.276	0.0	29,141,358	29,141,763
AW062792.1	SAMN00156993	Colon	688	A ↔ T	NM_001082480.2	8	zinc finger protein 623 (ZNF623)	671	97.914	0.0	3,041	3,707
AW062793.1	SAMN00156993	Colon	283	A ↔ T	NC_000007.14	7	PREDICTED: Homo sapiens speedy/RINGO cell cycle regulator family member E2B (SPDYE2B)	206	84.466	1.86e−49	102,357,109	102,357,313
AW062794.1	SAMN00156993	Colon	115	A ↔ T	NM_001282507.1	16	PKD1P4-NPIPA8 readthrough (PKD1P4-NPIPA8), non-coding RNA+ nuclear pore complex interacting protein family member A7 (NPIPA7)	98	96.939	2.43e−38	778	875
AW062796.1	SAMN00156993	Colon	196	A ↔ T	NM_001256410.1	10	RAB18, member RAS oncogene family (RAB18)	183	98.361	1.37e−85	876	1,058
AW062799.1	SAMN00156993	Colon	316	A ↔ T	NM_022130.3	5	golgi phosphoprotein 3 (GOLPH3), transcript variant X1, mRNA	276	98.551	1.27e−135	1,152	1,427
AW062801.1	SAMN00156993	Colon	263	A ↔ T	NM_020700.1	12	protein phosphatase, Mg2+/Mn2+ dependent 1H (PPM1H)	245	99.592	6.00e−124	4,901	4,657
AW062802.1	SAMN00156993	Colon	360	A ↔ T	NM_000610.3	11	CD44 molecule (Indian blood group) (CD44),	346	97.399	4.38e−165	2,799	2,454
AW062803.1	SAMN00156993	Colon	642	A ↔ T	NM_005814.2	1	glycoprotein A33 (GPA33), transcript variant X1, mRNA	351	92.877	3.51e−141	825	1,171
AW062804.1	SAMN00156993	Colon	640	A ↔ T	NM_000701.7	1	ATPase Na+/K+ transporting subunit alpha 1 (ATP1A1	636	96.855	0.0	3,394	2,760
AW062805.1	SAMN00156993	Colon	622	A ↔ T	NC_000009.12	9	golgi membrane protein 1 (GOLM1)	400	94.500	1.55e−174	86,074,944	86,075,342
AW062807.1*	SAMN00156993	Colon	616	A ↔ T	NM_001079524.1	4	phosphoribosylaminoimidazole carboxylase and phosphoribosylaminoimidazolesuccinocarboxamide synthase (PAICS)	582	88.144	0.0	1,737	2,276
AW062808.1	SAMN00156993	Colon	325	A ↔ T	NM_015658.3	2	NOC2 like nucleolar associated transcriptional repressor (NOC2L)	290	98.621	7.55e−143	953	1,242
AW062809.1	SAMN00156993	Colon	609	A ↔ T	NM_001278699.2	3	RNA polymerase II subunit H (POLR2H)	544	97.978	0.0	1,229	1,771
AW062815.1*	SAMN00156993	Colon	621	A ↔ T	NW_017852933.1	16	nuclear pore complex-interacting protein family member A7-like (LOC102724993)	606	98.515	0.0	1,699,909	1,699,305
AW062818.1	SAMN00156993	Colon	363	A ↔ T	NC_000003.12	3	coiled-coil-helix-coiled-coil-helix domain containing 6 (CHCHD6)	349	98.281	7.23e−173	126,914,442	126,914,789
AW062819.1	SAMN00156993	Colon	524	A ↔ T	NM_015658.3	1	NOC2 like nucleolar associated transcriptional repressor (NOC2L)	252	99.603	7.71e−128	991	1,242
AW062820.1*	SAMN00156993	Colon	508	A ↔ T	NM_001293298.1	15	cell migration inducing hyaluronidase 1 (CEMIP)	388	97.423	0.0	6,478	6,092
AW062821.1*	SAMN00156993	Colon	336	A ↔ T	NM_002295.5	3	ribosomal protein SA (RPSA)	329	95.441	9.69e−147	182	510
AW062822.1	SAMN00156993	Colon	350	A ↔ T	NM_007278.1	17	GABA type A receptor-associated protein (GABARAP)	155	96.774	1.09e−66	650	497
AW062823.1	SAMN00156993	Colon	619	A ↔ T	NM_001300832.2	11	FERM domain containing 8 (FRMD8)	499	98.998	0.0	2,751	2,253
AW062824.1	SAMN00156993	Colon	294	A ↔ T	NM_001731.2	12	BTG anti-proliferation factor 1 (BTG1), mRNA	289	97.924	1.63e−139	1,410	1,122
AW062825.1	SAMN00156993	Colon	358	A ↔ T	NM_002086.4	17	growth factor receptor bound protein 2 (GRB2), transcr	246	98.780	1.30e−120	2,793	3,038
AW062826.1	SAMN00156993	Colon	311	A ↔ T	NC_000007.14	7	PREDICTED: Homo sapiens speedy/RINGO cell cycle regulator family member E2B (SPDYE2B)	206	84.466	1.86e−49	102,357,109	102,357,313
AW062827.1	SAMN00156993	Colon	500	A ↔ T	NM_014455.3	1	ring finger protein 115 (RNF115)	380	99.474	0.0	1,064	685
AW062828.1	SAMN00156993	Colon	490	A ↔ T	NM_001271969.1	6	heat shock protein 90 alpha family class B member 1 (HSP90AB1),	364	98.077	1.54e−179	2,091	1,730
AW062829.1	SAMN00156993	Colon	588	A ↔ T	NM_001024216.2	1	filamin binding LIM protein 1 (FBLIM1)	525	99.048	0.0	2,354	2,877
AW062830.1	SAMN00156993	Colon	191	A ↔ T	NM_003302.2	7	thyroid hormone receptor interactor 6 (TRIP6)	173	96.532	4.98e−75	1,639	1,467
AW062831.1	SAMN00156993	Colon	157	A ↔ T	NM_003749.2	13	insulin receptor substrate 2 (IRS2), mRNA	144	94.444	5.12e−55	5,643	5,503
AW175732.1*	SAMN00156993	Colon	392	A ↔ T	NC_000015.10	15	cell migration inducing hyaluronidase 1 (CEMIP)	237	96.203	1.72e−104	80,878,828	80,879,064
AW175733.1	SAMN00156993	Colon	748	A ↔ T	NM_032833.4	1	protein phosphatase 1 regulatory subunit 15B (PPP1R15B)	574	95.645	0.0	71	643
AW175736.1	SAMN00156993	Colon	391	A ↔ T	NM_005080.3	22	X-box binding protein 1 (XBP1)	284	97.183	2.13e−133	497	779
AW175738.1*	SAMN00156993	Colon	195	A ↔ T	NC_000003.12	3	chromosome 3 open reading frame 14 (C3orf14)	137	95.620	6.63e−54	62,332,406	62,332,270
AW175740.1*	SAMN00156993	Colon	382	A ↔ T	NC_000008.11	8	pericentriolar material 1 (PCM1)	288	97.917	2.11e−138	17,968,415	17,968,700
AW175741.1	SAMN00156993	Colon	277	A ↔ T	NC_000002.12	2	phosphodiesterase 11A (PDE11A)	203	95.567	3.80e−86	177,849,206	177,849,004
AW175742.1	SAMN00156993	Colon	701	A ↔ T	NC_000001.11	1	nuclear receptor subfamily 5 (NR5A2)	190	98.421	1.75e−89	200,040,067	200,040,255
AW175747.1*	SAMN00156993	Colon	182	A ↔ T	NC_000012.12	12	ribosomal protein SA pseudogene 52 (RPSAP52)	122	92.623	5.23e−40	65,765,193	65,765,313
AW175749.1	SAMN00156993	Colon	379	A ↔ T	NC_000003.12	2	tigger transposable element also zinc finger protein 621 (ZNF621) derived 1 (TIGD1)	244	98.361	7.82e−118	3,165,299	3,165,056
AW175751.1	SAMN00156993	Colon	403	A ↔ T	NT_187585.1	11	potassium voltage-gated channel subfamily Q member 1 (KCNQ1)	336	94.048	4.54e−140	49,605	49,278
AW175753.1	SAMN00156993	Colon	800	A ↔ T	NC_000005.10	5	MCC regulator of WNT signaling pathway (MCC)	140	84.286	6.87e−29	113,144,132	113,144,267
AW176932.1*	SAMN00156993	Colon	655	A ↔ T	NM_001079524.1	4	phosphoribosylaminoimidazole carboxylase and phosphoribosylaminoimidazolesuccinocarboxamide synthase (PAICS)	624	97.917	0.0	2,426	1,803
AW176933.1*	SAMN00156993	Colon	286	A ↔ T	NC_000012.12	12	ATPase sarcoplasmic/endoplasmic reticulum Ca2+ transporting 2	283	97.173	3.56e−131	110,285,901	110,285,620
AW176934.1*	SAMN00156993	Colon	294	A ↔ T	NC_000012.12	12	ATPase sarcoplasmic/endoplasmic reticulum Ca2+ transporting 2	285	99.649	3.49e−146	110,285,904	110,285,620
AW176938.1	SAMN00156993	Colon	183	A ↔ T	NM_014280.2	1	DnaJ heat shock protein family (Hsp40) member C8 (DNAJC8),	179	96.648	6.40e−79	661	483
AW176940.1	SAMN00156993	Colon	334	A ↔ T	NC_000014.9	14	serine palmitoyltransferase long chain base subunit 2 (SPTLC2)	306	98.693	2.68e−152	77,544,607	77,544,912
AW176941.1	SAMN00156993	Colon	664	A ↔ T	NC_000002.12	2	MIR4435-2 host gene (MIR4435-2HG)	580	98.276	0.0	111,301,721	111,302,299
AW176942.1	SAMN00156993	Colon	337	A ↔ T	NM_001265603.1	15	mortality factor 4 like 1 (MORF4L1)	279	98.208	4.57e−135	565	843
AW176943.1	SAMN00156993	Colon	627	A ↔ T	NC_000016.10	16	membrane bound transcription factor peptidase, site 1(MBTPS1)	500	98.200	0.0	84,061,142	84,061,638
AW176945.1	SAMN00156993	Colon	361	A ↔ T	NM_030629.2	16	c-Maf inducing protein (CMIP)	326	98.466	9.49e−162	2,775	2,450
AW176946.1	SAMN00156993	Colon	664	A ↔ T	NM_001873.3	4	carboxypeptidase E (CPE)	307	98.046	5.79e−149	1,193	890
AW176947.1	SAMN00156993	Colon	680	A ↔ T	NC_000004.12	4	huntingtin (HTT)	660	98.485	0.0	3,200,416	3,201,074
AW176948.1	SAMN00156993	Colon	360	A ↔ T	NM_001256799.2	12	glyceraldehyde-3-phosphate dehydrogenase (GAPDH)	311	99.357	2.05e−158	831	1,140
AW176949.1	SAMN00156993	Colon	304	A ↔ T	NM_005731.3	2	actin related protein 2/3 complex subunit 2 (ARPC2)	272	94.118	7.87e−113	867	597
AW176952.1	SAMN00156993	Colon	629	A ↔ T	NC_000002.12	2	integrin subunit alpha 6 (ITGA6)	630	99.841	0.0	172,430,001	172,429,372
AW176953.1	SAMN00156993	Colon	375	A ↔ T	NM_001204510.1	17	SENP3-EIF4A1 readthrough (NMD candidate) (SENP3-EIF4A1), long non-coding RNA+eukaryotic translation initiation factor 4A1 (EIF4A1)	338	99.408	5.59e−174	824	487
AW176954.1	SAMN00156993	Colon	282	A ↔ T	NM_006761.4	17	tyrosine 3-monooxygenase/tryptophan 5-monooxygenase activation protein epsilon (YWHAE),non-coding RNA	264	98.485	2.14e−128	961	699
AW176955.1	SAMN00156993	Colon	470	A ↔ T	NC_000007.14	10	general transcription factor IIi	264	100.000	3.54e−136	74,722,574	74,722,311
AW176958.1	SAMN00156993	Colon	255	A ↔ T	NM_001291484.2	19	carcinoembryonic antigen related cell adhesion molecule 5 (CEACAM5)	227	99.119	1.02e−111	1,130	1,356
AW176959.1*	SAMN00156993	Colon	682	A ↔ T	NM_001402.5	6	eukaryotic translation elongation factor 1 alpha 1 (EEF1A1)	647	92.427	0.0	923	1,566
AW176960.1*	SAMN00156993	Colon	622	A ↔ T	NM_001402.5	6	eukaryotic translation elongation factor 1 alpha 1 (EEF1A1)	610	99.672	0.0	891	1,499
AW176962.1	SAMN00156993	Colon	309	A ↔ T	NM_005514.7	6	major histocompatibility complex, class I, B (HLA-B)	276	97.464	1.28e−130	1,338	1,063
AW176963.1*	SAMN00156993	Colon	451	A ↔ T	NC_000001.11	1	erythrocyte membrane protein band 4 (EPB41)	381	98.163	0.0	29,114,294	29,113,915
AW176964.1*	SAMN00156993	Colon	392	A ↔ T	NC_000001.11	1	erythrocyte membrane protein band 4 (EPB41)	381	98.688	0.0	29,114,294	29,113,915
AW176965.1	SAMN00156993	1Colon	628	A ↔ T	NM_001137604.2	2	RNA polymerase I subunit B (POLR1B)	421	99.050	0.0	3,159	3,579
AW176968.1	SAMN00156993	Colon	633	A ↔ T	NM_001172663.1	16	RAB40C, member RAS oncogene family (RAB40C)	160	98.750	1.79e−74	1,801	1,643
AW176969.1	SAMN00156993	Colon	348	A ↔ T	NM_002588.3	5	protocadherin gamma subfamily B, 3 (PCDHGB3)	350	97.714	1.57e−169	4,400	4,051
AW176971.1*	SAMN00156993	Colon	650	A ↔ T	NM_001402.5	6	eukaryotic translation elongation factor 1 alpha 1 (EEF1A1)	307	98.697	7.44e−153	1,305	1,000
AW176972.1	SAMN00156993	Colon	374	A ↔ T	NM_001167671.2	3	LIM domain containing preferred translocation partner in lipoma (LPP)	369	98.103	0.0	16,859	17,225
AW176973.1	SAMN00156993	Colon	645	A ↔ T	NC_000016.10	16	zinc and ring finger 1 (ZNRF1)	587	98.978	0.0	75,058,197	75,058,783
AW176974.1	SAMN00156993	Colon	652	A ↔ T	NM_001276418.1	9	SEC16 homolog A, endoplasmic reticulum export factor (SEC16A)	585	99.316	0.0	8,695	8,111
AW176982.1*	SAMN00156993	Colon	560	A ↔ T	NC_012920.1	mito	cox1	513	99.220	0.0	6,537	6,025
AW903505.1*	SAMN00159106	Nervous_normal	436	A → T → C → G → A	NR_148543.1	2	clathrin heavy chain linker domain containing 1 (CLHC1)	437	98.169	0.0	569	1,001
AW903508.1	SAMN00159106	Nervous_normal	443	A → T → C → G → A	NM_015018.3	6	DOP1 leucine zipper like protein A (DOP1A)	362	99.448	0.0	7,625	7,264
AW903509.1*	SAMN00159106	Nervous_normal	449	A → T → C → G → A	NM_001351724.1	4	Rap guanine nucleotide exchange factor 2 (RAPGEF2)	451	97.561	0.0	5,954	6,400
AW903510.1	SAMN00159106	Nervous_normal	472	A → T → C → G → A	NM_004859.3	17	clathrin heavy chain (CLTC)	406	99.015	0.0	3,799	4,202
AW903511.1	SAMN00159106	Nervous_normal	464	A → T → C → G → A	NC_000014.9	14	endoplasmic reticulum oxidoreductase 1 alpha (ERO1A)	444	97.973	0.0	52,686,677	52,687,114
AW903512.1*	SAMN00159106	Nervous_normal	185	A → T → C → G → A	NM_024996.5	3	G elongation factor mitochondrial 1 (GFM1)	186	90.323	9.61e−61	3,446	3,264
AW903513.1*	SAMN00159106	Nervous_normal	254	A → T → C → G → A	NM_024996.5	3	G elongation factor mitochondrial 1 (GFM1)	166	100.000	2.60e−81	3,237	3,072
AW903514.1*	SAMN00159106	Nervous_normal	423	A → T → C → G → A	NM_001351724.1	4	Rap guanine nucleotide exchange factor 2 (RAPGEF2)	424	99.057	0.0	5,962	6,384
AW903515.1	SAMN00159106	Nervous_normal	447	A → T → C → G → A	NC_000008.11	8	mitochondrial calcium uptake family member 3	447	100.000	0.0	17,028,849	17,028,403
AW903516.1*	SAMN00159106	Nervous_normal	446	A → T → C → G → A	NM_007353.2	7	G protein subunit alpha 12 (GNA12)	409	99.511	0.0	3,841	4,249
AW903517.1*	SAMN00159106	Nervous_normal	382	A → T → C → G → A	NM_004529.3	9	MLLT3, super elongation complex subunit (MLLT3)	379	99.736	0.0	4,755	4,377
AW903518.1*	SAMN00159106	Nervous_normal	261	A → T → C → G → A	NC_012920.1	mito	cox1	133	99.248	9.61e−61	6,634	6,766
AW903519.1*	SAMN00159106	Nervous_normal	457	A → T → C → G → A	NC_000001.11	1	membrane associated guanylate kinase (MAGI3)	443	97.065	0.0	113,403,203	113,403,645
AW903520.1	SAMN00159106	Nervous_normal	461	A → T → C → G → A	NR_029435.1	14	PSMA3 antisense RNA 1 (PSMA3-AS1), long non-coding RNA	447	100.000	0.0	2,426	1,980
AW903521.1*	SAMN00159106	Nervous_normal	449	A → T → C → G → A	NC_012920.1	mito	cox1	451	98.670	0.0	6,534	6,982
AW903523.1	SAMN00159106	Nervous_normal	447	A → T → C → G → A	NC_000004.12	4	NEDD4 binding protein 2 (N4BP2)	445	99.775	0.0	40,063,375	40,063,375
AW903524.1*	SAMN00159106	Nervous_normal	455	A → T → C → G → A	NM_001286691.1	9	MLLT3, super elongation complex subunit (MLLT3)	449	100.000	0.0	4,671	4,223
AW903525.1	SAMN00159106	Nervous_normal	431	A → T → C → G → A	NC_000003.12	3	C-X9-C motif containing 1 (269)	269	99.628	2.40e−136	28,242,970	28,242,702
AW903526.1*	SAMN00159106	Nervous_normal	466	A → T → C → G → A	NR_029435.1	14	PSMA3 antisense RNA 1 (PSMA3-AS1) and long non-coding RNA	456	99.781	0.0	2,426	1,971
AW903527.1*	SAMN00159106	Nervous_normal	446	A → T → C → G → A	NM_004529.3	9	MLLT3, super elongation complex subunit (MLLT3)	446	99.776	0.0	4,213	4,658
AW903528.1*	SAMN00159106	Nervous_normal	461	A → T → C → G → A	NR_148543.1	2	clathrin heavy chain linker domain containing 1 (CLHC1)	457	99.344	0.0	1,034	578
AW903529.1	SAMN00159106	Nervous_normal	464	A → T → C → G → A	NR_049782.1	2	sorting nexin 17 (SNX17)	455	98.901	0.0	1,025	573
AW903530.1	SAMN00159106	Nervous_normal	418	A → T → C → G → A	NM_004800.2	13	transmembrane 9 superfamily member 2 (TM9SF2)	395	98.734	0.0	2,509	2,115
AW903531.1	SAMN00159106	Nervous_normal	408	A → T → C → G → A	NC_000022.11	22	phosphatidylinositol 4-kinase alpha (PI4KA)	407	97.543	0.0	20,799,225	20,799,629
AW903532.1	SAMN00159106	Nervous_normal	279	A → T → C → G → A	NC_000003.12	3	zinc finger protein 148 (ZNF148)	194	98.969	5.50e−93	125,335,569	125,335,762
AW903533.1*	SAMN00159106	Nervous_normal	473	A → T → C → G → A	NM_001293092.1	7	G protein subunit alpha 12 (GNA12)	409	99.267	0.0	3,790	4,198
AW903534.1*	SAMN00159106	Nervous_normal	469	A → T → C → G → A	NC_000007.14	7	ataxin 7 like 1 (ATXN7L1)	461	99.132	0.0	105,869,501	105,869,961
AW903537.1	SAMN00159106	Nervous_normal	448	A → T → C → G → A	NC_000016.10	16	RNA binding fox-1 homolog 1 (RBFOX1)	431	99.304	0.0	6,537,483	6,537,913
AW903538.1	SAMN00159106	Nervous_normal	454	A → T → C → G → A	NC_000023.11	X	euroligin 4 X-linked (NLGN4X)	454	99.559	0.0	6,115,554	6,115,102
AW903539.1*	SAMN00159106	Nervous_normal	321	A → T → C → G → A	NC_000011.10	11	chromosome 11 open reading frame 80 (C11orf80)	248	93.952	9.08e−101	66,766,914	66,767,161
AW903540.1	SAMN00159106	Nervous_normal	329	A → T → C → G → A	NC_000007.14	7	family with sequence similarity 221 member A (FAM221A)	329	100.000	6.36e−172	23,695,685	23,696,013
AW903541.1*	SAMN00159106	Nervous_normal	470	A → T → C → G → A	NC_000005.10	5	teneurin transmembrane protein 2 (TENM2)	455	99.341	0.0	167,499,052	167,499,506
AW903542.1*	SAMN00159106	Nervous_normal	464	A → T → C → G → A	NM_024996.5	3	G elongation factor mitochondrial 1 (GFM1)	455	99.560	0.0	2,980	3,434
AW903543.1	SAMN00159106	Nervous_normal	470	A → T → C → G → A	NC_000001.11	1	vacuolar protein sorting 72 homolog (VPS72)	468	99.359	0.0	151,182,693	151,183,160
AW903544.1*	SAMN00159106	Nervous_normal	458	A → T → C → G → A	NM_018947.5	7	cytochrome c, somatic (CYCS), mRNA	460	99.130	0.0	703	245
AW903545.1*	SAMN00159106	Nervous_normal	458	A → T → C → G → A	NC_000005.10	5	teneurin transmembrane protein 2 (TENM2)	459	99.782	0.0	167,499,058	167,499,516
AW903546.1	SAMN00159106	Nervous_normal	236	A → T → C → G → A	NC_000011.10	11	GRB2 associated binding protein 2 (GAB2)	154	97.403	2.05e−67	78,340,987	78,341,140
AW903547.1*	SAMN00159106	Nervous_normal	441	A → T → C → G → A	NC_000001.11	1	membrane associated guanylate kinase (MAGI3)	427	97.190	0.0	113,403,203	113,403,629
AW903550.1	SAMN00159106	Nervous_normal	460	A → T → C → G → A	NM_172350.2	1	CD46 molecule (CD46)	462	99.134	0.0	2,971	2,510
AW903551.1*	SAMN00159106	Nervous_normal	215	A → T → C → G → A	NC_012920.1	mito	cox1	109	99.083	2.11e−47	7,416	7,308
AW903553.1	SAMN00159106	Nervous_normal	468	A → T → C → G → A	NM_001351562.1	9	TLE family member 4 (TLE4)	469	97.655	0.0	4176	4643
AW903554.1*	SAMN00159106	Nervous_normal	357	A → T → C → G → A	NM_018947.5	7	cytochrome c, somatic (CYCS), mRNA	355	99.155	0.0	682	328
AW903556.1*	SAMN00159106	Nervous_normal	444	A → T → C → G → A	NM_004529.3	9	MLLT3, super elongation complex subunit (MLLT3)	446	99.327	0.0	4,746	4,301
AW903557.1	SAMN00159106	Nervous_normal	458	A → T → C → G → A	NC_000012.12	12	synaptotagmin 1 (SYT1)	462	98.701	0.0	79,289,394	79,288,933
AW903558.1	SAMN00159106	Nervous_normal	476	A → T → C → G → A	NC_000012.12	12	kinesin family member 21A (KIF21A)	477	99.161	0.0	39,406,699	39,407,174
AW903559.1	SAMN00159106	Nervous_normal	453	A → T → C → G → A	NC_000017.11	17	dynein axonemal heavy chain 17 (DNAH17)	290	99.310	6.59e−147	78,428,317	78,428,606
AW903560.1	SAMN00159106	Nervous_normal	459	A → T → C → G → A	NC_000006.12	6	capping protein regulator and myosin 1 linker 1 (CARMIL1)	403	96.030	0.0	25,338,780	25,338,379
AW903561.1*	SAMN00159106	Nervous_normal	463	A → T → C → G → A	NC_000014.9	14	PSMA3 antisense RNA 1 (PSMA3-AS1) and long non-coding RNA	449	98.886	0.0	58,265,829	58,265,381
AW903563.1*	SAMN00159106	Nervous_normal	427	A → T → C → G → A	NM_001352634.1	8	pericentriolar material 1 (PCM1)	423	99.291	0.0	5,954	6,376
AW903564.1	SAMN00159106	Nervous_normal	457	A → T → C → G → A	NM_025054.4	8	valosin containing protein interacting protein 1 (VCPIP1)	374	99.198	0.0	7,603	7,976
AW903565.1*	SAMN00159106	Nervous_normal	434	A → T → C → G → A	NC_000007.14	7	ataxin 7 like 1 (ATXN7L1)	427	99.297	0.0	105,869,531	105,869,956
AW903566.1	SAMN00159106	Nervous_normal	283	A → T → C → G → A	NM_015541.2	3	leucine rich repeats and immunoglobulin like domains 1 (LRIG1)	190	98.421	1.54e−88	3,447	3,258
AW903567.1	SAMN00159106	Nervous_normal	444	A → T → C → G → A	NC_000022.11	22	RNA binding fox-1 homolog 2 (RBFOX2)	344	92.442	3.11e−135	35,867,779	35,868,118
AW903568.1	SAMN00159106	Nervous_normal	428	A → T → C → G → A	NM_020755.3	6	serine incorporator 1 (SERINC1)	326	99.080	1.07e−164	2,332	2,008
AW903570.1	SAMN00159106	Nervous_normal	454	A → T → C → G → A	NT_187614.1	17	aminopeptidase puromycin sensitive pseudogene (NPEPPS1)	200	97.000	1.54e−88	2,236,540	2,236,343
AW903571.1	SAMN00159106	Nervous_normal	480	A → T → C → G → A	NM_001242866.2	21	protein arginine methyltransferase 2 (PRMT2)	297	99.663	1.82e−152	1,248	952
AW903573.1*	SAMN00159106	Nervous_normal	336	A → T → C → G → A	NC_012920.1	mito	cox1	236	98.729	1.15e−114	7,112	7,347
AW903576.1	SAMN00159106	Nervous_normal	472	A → T → C → G → A	NM_203401.1	4	stathmin 1 (STMN1)	468	98.932	0.0	361	827
AW903577.1	SAMN00159106	Nervous_normal	466	A → T → C → G → A	NC_000007.14	7	LUC7 like 2 (LUC7L2), pre-mRNA splicing factor	461	98.915	0.0	139,362,483	139,362,023
BE085177.1*	SAMN00159564	Breast	269	A → T → C → G → A	NC_000010.11	10	protein tyrosine phosphatase receptor type E (PTPRE)	242	98.347	6.87e−117	127,960,155	127,959,914
BE085178.1	SAMN00159564	Breast	176	A → T → C → G → A	NM_015888.4	1	hook microtubule tethering protein 1 (HOOK1)	171	92.982	2.67e−61	2,185	2,024
BE085183.1	SAMN00159564	Breast	293	A → T → C → G → A	NC_000010.11	10	SMC5-SMC6 complex localization factor 2 (SLF2)	92	95.652	5.99e−33	100,947,017	100,947,107
BE085186.1*	SAMN00159564	Breast	302	A → T → C → G → A	NM_002840.4	1	protein tyrosine phosphatase, receptor type F (PTPRF)	268	96.642	2.45e−121	2,985	2,724
BE085188.1	SAMN00159564	Breast	280	A → T → C → G → A	NM_007042.4	3	ribonuclease P/MRP subunit p14 (RPP14)	193	96.373	1.55e−83	328	139
BE085197.1	SAMN00159564	Breast	196	A → T → C → G → A	NM_001281724.2	3	biotinidase (BTD)	106	99.057	2.73e−46	2,275	2,170
BE085202.1	SAMN00159564	Breast	170	A → T → C → G → A	NM_021009.6	12	ubiquitin C (UBC)	102	89.216	1.01e−25	1,869	1,772
BE145238.1	SAMN00157869	Head_neck	333	A ↔ T	NM_001042465.2	10	prosaposin (PSAP)	334	97.904	1.50e−163	1,739	2,072
BE145239.1*	SAMN00157869	Head_neck	293	A ↔ T	NM_001104629.1	4	chromosome 4 open reading frame 19 (C4orf19)	292	97.260	5.59e−138	1,828	2,119
BE145240.1	SAMN00157869	Head_neck	169	A ↔ T	NM_001033925.1	10	TIA1 cytotoxic granule associated RNA binding protein like 1 (TIAL1)	169	96.450	4.73e−74	1,013	1,181
BE145242.1	SAMN00157869	Head_neck	452	A ↔ T	NM_022470.3	3	zinc finger matrin-type 3 (ZMAT3)	404	98.267	0.0	6,273	6,675
BE145243.1	SAMN00157869	Head_neck	110	A ↔ T	NM_001101662.1	1	nardilysin convertase (NRDC)	110	99.091	1.76e−48	1,445	1,336
BE145245.1	SAMN00157869	Head_neck	421	A ↔ T	NC_000005.10	5	small integral membrane protein 3 (SMIM3)	416	97.837	0.0	150,784,346	150,784,758
BE145246.1	SAMN00157869	Head_neck	266	A ↔ T	NM_001033047.2	4	nephronectin (NPNT)	267	92.884	9.75e−106	391	656
BE145247.1	SAMN00157869	Head_neck	359	A ↔ T	NW_019805496.1	11	mitochondrial carrier 2 (MTCH2)	356	96.067	5.39e−163	55,682	56,035
BE145248.1*	SAMN00157869	Head_neck	247	A ↔ T	NC_000014.9	14	valosin containing protein lysine methyltransferase (VCPKMT)	240	92.083	9.96e−91	50,108,378	50,108,617
BE145250.1*	SAMN00157869	Head_neck	289	A ↔ T	NC_000014.9	14	valosin containing protein lysine methyltransferase (VCPKMT)	290	95.517	1.58e−128	50,108,285	50,108,574
BE145251.1	SAMN00157869	Head_neck	329	A ↔ T	NC_000009.12	9	spectrin alpha, non-erythrocytic 1 (SPTAN1)	330	96.061	9.15e−151	128,610,482	128,610,155
BF332836.1	SAMN00160205	Breast	298	A → T → C → G → A	NC_000001.11	1	putative homeodomain transcription factor 1 (PHTF1)	230	95.652	4.22e−99	113,710,355	113,710,126
BF332837.1	SAMN00160205	Breast	376	A → T → C → G → A	NC_000002.12	2	BCL2 like 11(BCL2 L11)	241	98.340	3.20e−115	111,159,962	111,160,199
BF332838.1	SAMN00160205	Breast	359	A → T → C → G → A	NC_000008.11	8	integrator complex subunit 9 (INTS9)	237	97.468	1.16e−109	28,784,748	28,784,981
BF332840.1	SAMN00160205	Breast	300	A → T → C → G → A	NM_018364.4	1	round spermatid basic protein 1 (RSBN1)	209	94.737	1.20e−84	3,267	3,062
BF332841.1	SAMN00160205	Breast	372	A → T → C → G → A	NM_002266.3	17	karyopherin subunit alpha 2 (KPNA2)	269	96.654	6.82e−122	2,022	1,757
BF332842.1	SAMN00160205	Breast	378	A → T → C → G → A	NM_003761.4	2	vesicle associated membrane protein 8 (VAMP8)	253	99.209	8.76e−126	296	548
BF332846.1	SAMN00160205	Breast	280	A → T → C → G → A	NM_017409.3	12	homeobox C10 (HOXC10)	190	95.789	4.35e−79	1,958	1,773
BF332847.1	SAMN00160205	Breast	405	A → T → C → G → A	NC_000007.14	7	3-hydroxyisobutyrate dehydrogenase (HIBADH)	275	97.818	2.42e−131	27,629,869	27,629,596
BF332849.1	SAMN00160205	Breast	308	A → T → C → G → A	NC_000014.9	14	dishevelled associated activator of morphogenesis 1 (DAAM1)	206	95.631	1.99e−87	59,268,775	59,268,570
BF332850.1	SAMN00160205	Breast	383	A → T → C → G → A	NC_000012.12	12	spermatogenesis associated serine rich 2 (SPATS2)	296	96.284	4.02e−134	49,458,046	49,457,752
BF332851.1	SAMN00160205	Breast	335	A → T → C → G → A	NC_000013.11	13	SMAD family member 9 (SMAD9)	249	98.394	8.83e−121	36,916,214	36,916,462
BF332854.1	SAMN00160205	Breast	293	A → T → C → G → A	NC_000003.12	3	coiled-coil domain containing 66 (CCDC66)	221	98.190	4.19e−104	56,561,670	56,561,451
BF332855.1	SAMN00160205	Breast	309	A → T → C → G → A	NC_000014.9	14	RAD51 paralog B (RAD51B)	222	96.396	1.97e−97	68,178,422	68,178,203
BF353453.1	SAMN00161174	Head_neck	395	A ↔ T	NM_002274.3	17	keratin 13 (KRT13)	53	100.000	8.30e−19	1,679	1,627
BF354529.1*	SAMN00160318	Head neck	397	A → T → C → G → A	NM_015596.2	19	kallikrein related peptidase 13 (KLK13)	317	98.107	1.09e−154	1,348	1,664
BF354541.1	SAMN00160318	Head neck	363	A → T → C → G → A	NM_030634.2	1	zinc finger protein 436 (ZNF436)	295	98.644	8.52e−146	3,915	4,209
BF354544.1	SAMN00160318	Head neck	449	A → T → C → G → A	NC_000006.12	6	protein phosphatase 1 regulatory subunit 10 (PPP1R10)	384	98.698	0.0	30,600,715	30,601,097
BF354553.1	SAMN00160318	Head neck	387	A → T → C → G→ A	NW_016107297.1	5	GUSB Pseudogene (GUSBP1)	332	98.193	1.79e−162	163,285	162,954
BF354559.1	SAMN00160318	Head neck	193	A → T → C → G → A	XM_011524110.3	17	small G protein signaling modulator 2 (SGSM2)	99	93.939	5.99e−33	2,457	2,360
BF354564.1*	SAMN00160318	Head neck	402	A → T → C → G → A	NM_015596.2	19	kallikrein related peptidase 13 (KLK13)	306	98.366	1.09e−149	1,359	1,664
BF354572.1	SAMN00160318	Head neck	389	A → T → C → G → A	XM_011514699.2	6	glucose-fructose oxidoreductase domain containing 1 (GFOD1)	346	98.844	3.80e−174	3,008	3,353
BF370229.1	SAMN00160862	Prostate_normal	282	A → T → C → G → A	NM_000138.4	15	fibrillin 1 (FBN1)	127	95.276	5.87e−48	8,263	8,388
BF370230.1	SAMN00160862	Prostate_normal	282	A → T → C → G → A	NM_006887.4	15	ZFP36 ring finger protein like 2 (ZFP36L2)	346	97.399	3.85e−164	2,544	2,201
BF370232.1	SAMN00160862	Prostate_normal	410	A → T → C → G → A	NC_012920.1	mito	atp6	359	98.329	6.31e−177	8,663	9,020
BF370233.1	SAMN00160862	Prostate_normal	437	A → T → C → G → A	NC_000023.11	x	ubiquitin specific peptidase 9 X-linked (USP9X)	373	98.928	0.0	41,186,360	41,185,989
BF370234.1	SAMN00160862	Prostate_normal	413	A → T → C → G → A	NC_000012.12	12	lysine demethylase 5A (KDM5A)	349	97.994	2.96e−170	326,420	326,075
BF370235.1	SAMN00160862	Prostate_normal	294	A → T → C → G → A	NM_001288733.1	17	testis expressed 2 (TEX2)	127	98.425	9.68e−56	4,533	4,659
BF370236.1	SAMN00160862	Prostate_normal	294	A → T → C → G → A	NC_000009.12	9	basonuclin 2 (BNC2)	194	98.969	5.50e−93	16,514,557	16,514,750
BF370237.1	SAMN00160862	Prostate_normal	423	A → T → C → G → A	NM_001146227.1	8	ribosomal protein S20 (RPS20)	304	99.013	6.54e−152	493	191
BF370238.1	SAMN00160862	Prostate_normal	332	A → T → C → G → A	NC_000004.12	4	sec1 family domain containing 2 (SCFD2)	243	98.354	2.47e−116	52,874,276	52,874,518
BF370239.1	SAMN00160862	Prostate_normal	355	A → T → C → G → A	NM_018046.4	5	angiogenic factor with G-patch and FHA domains 1 (AGGF1)	293	98.294	1.43e−143	4,058	4,350
BF370241.1	SAMN00160862	Prostate_normal	305	A → T → C → G → A	NC_000017.11	17	tubulin folding cofactor D (TBCD)	173	98.844	2.60e−81	82,761,458	82,761,630
BF370242.1	SAMN00160862	Prostate_normal	250	A → T → C → G → A	NC_000001.11	1	GNG12, DIRAS3 and WLS antisense RNA 1 (GNG12-AS1) and Wnt ligand secretion mediator (WLS)	127	98.425	9.68e−56	68,182,918	68,182,792
BF370243.1	SAMN00160862	Prostate_normal	413	A → T → C → G → A	NC_000005.10	5	ubiquitin conjugating enzyme E2 D2 (UBE2D2)	272	97.059	8.76e−126	139,610,960	139,610,690
BF798643.1	SAMN00162090	Colon_ins	420	A ↔ T	NR_045104.1	15	integrator complex subunit 14 (INTS14)	290	92.759	5.75e−114	163	448
BF798647.1	SAMN00162090	Colon_ins	415	A ↔ T	NC_012920.1	mito	12s	271	94.096	3.46e−111	915	649
BF798651.1	SAMN00162090	Colon_ins	363	A ↔ T	NM_032593.2	9	histidine triad nucleotide binding protein 2 (HINT2)	57	94.737	5.12e−15	429	484
BF798653.1	SAMN00162090	Colon_ins	425	A ↔ T	NC_012920.1	mito	12s	270	94.815	5.75e−114	649	915
BF798654.1	SAMN00162090	Colon_ins	375	A ↔ T	NM_001291920.1	9	retinoid X receptor alpha (RXRA)	136	95.588	8.10e−53	2,040	2,170
BF798660.1	SAMN00162090	Colon_ins	464	A ↔ T	NC_012920.1	mito	12s	269	97.398	1.22e−125	649	915
BF798665.1	SAMN00162090	Colon_ins	485	A ↔ T	NM_004069.4	19	adaptor related protein complex 2 sigma 1 subunit (AP2S1)	373	98.123	0.0	564	193
BF798667.1	SAMN00162090	Colon_ins	521	A ↔ T	NM_005561.3	13	lysosomal associated membrane protein 1 (LAMP1)	481	95.842	0.0	392	872

Genes corresponding to more than one EST sequence are indicated by *.

**Table 2:** List of ESTs based on the genes identified in more than one biosample.

EST Acc No.	Biosample	Cancer tissue	Length of EST	Swinger transformation	Acc. No. of aligned sequence	Chrom-osome	Gene	Aligned Seq. length	%ID	evalue	5'	3'
BE085181.1	SAMN00159564	Breast	109	A → T → C → G → A	NC_012920.1	mito	16s	41	95.122	6.21e−08	2,885	2,924
BE085203.1	SAMN00159564	Breast	282	A → T → C → G → A	NC_012920.1	mito	16s	189	98.413	1.54e−88	1,950	1,764
BE085205.1	SAMN00159564	Breast	134	A → T → C → G → A	NC_012920.1	mito	16s	89	97.753	1.29e−34	2,882	2,794
BE085207.1	SAMN00159564	Breast	245	A → T → C → G → A	NC_012920.1	mito	16s	185	97.838	1.20e−84	2,838	3,021
BF332839.1	SAMN00160205	Breast	249	A → T → C → G → A	NC_012920.1	mito	16s	156	97.436	5.70e−68	2,258	2,105
BF798658.1	SAMN00162090	Colon_ins	624	A ↔ T	NC_012920.1	mito	16s+trnaL+ND1	464	98.276	0.0	3,075	3,537
BF798678.1	SAMN00162090	Colon_ins	589	A ↔ T	NC_012920.1	mito	16s+trnaL+ND1	464	97.414	0.0	3,075	3,537
AI939948.1	SAMN00156461	Colon	529	A ↔ T	NM_000476.2	9	adenylate kinase 1 (AK1)	504	97.024	0.0	198	700
AI940588.1	SAMN00156483	Head_neck	650	A ↔ T	NM_001318121.1	9	adenylate kinase 1 (AK1)	574	98.606	0.0	177	749
BF332844.1	SAMN00160205	Breast	251	A → T → C → G → A	NM_000100.3	21	cystatin B (CSTB)	170	96.471	7.32e−72	452	287
AW176935.1	SAMN00156993	Colon	386	A ↔ T	NC_000021.9	21	cystatin b (CSTB)	349	99.140	2.58e−177	43,774,237	43,774,583
AW176936.1	SAMN00156993	Colon	368	A ↔ T	NC_000021.9	21	cystatin b (CSTB)	348	99.425	5.55e−179	43,774,237	43,774,583
AW903549.1	SAMN00159106	Nervous_normal	444	A → T → C → G → A	NM_001308390.1	18	DLG associated protein 1 (DLGAP1)	446	99.327	0.0	4,908	4,464
AW176970.1	SAMN00156993	Colon	701	A ↔ T	NC_000020.11	20	DLG associated protein 4 (DLGAP4)	685	98.686	0.0	36,525,990	36,526,674
BF332852.1	SAMN00160205	Breast	406	A → T → C → G → A	NM_153201.3	11	heat shock protein family A (Hsp70) member 8 (HSPA8)	302	97.351	1.84e−142	925	626
BF798649.1	SAMN00162090	Colon_ins	529	A ↔ T	NM_006597.5	11	heat shock protein family A (Hsp70) member 8 (HSPA8)	486	95.062	0.0	386	865
AW062795.1	SAMN00156993	Colon	639	A ↔ T	NM_005120.2	X	mediator complex subunit 12 (MED12)	502	99.801	0.0	4,475	3,974
BF332843.1	SAMN00160205	Breast	340	A → T → C → G → A	NM_005121.2	17	mediator complex subunit 13 (MED13)	214	98.131	9.08e−101	943	731
BF332848.1	SAMN00160205	Breast	372	A → T → C → G → A	NM_001271811.1	12	mediator complex subunit 21 (MED21)	245	97.959	2.47e−116	1,097	854
AW176957.1	SAMN00156993	Colon	517	A ↔ T	NC_012920.1	mito	nd1	495	99.192	0.0	3,663	4,157
BF798657.1	SAMN00162090	Colon_ins	363	A ↔ T	NC_012920.1	mito	nd1	210	93.333	1.30e−80	3,333	3,537
AW903507.1	SAMN00159106	Nervous_normal	215	A → T → C → G → A	NC_012920.1	mito	nd2	214	99.533	2.51e−106	4,739	4,952
BF332853.1	SAMN00160205	Breast	308	A → T → C → G → A	NC_012920.1	mito	nd2	216	99.074	9.02e−106	4,724	4,939
BF354531.1	SAMN00160318	Head neck	373	A → T → C → G → A	NC_012920.1	mito	nd2	242	98.760	1.48e−118	4,482	4,723
BF354537.1	SAMN00160318	Head neck	345	A → T → C → G → A	NC_012920.1	mito	nd2	232	100.000	5.31e−118	4,492	4,723
BI032899.1	SAMN00162655	Nervous_normal	424	A ↔ C	NC_012920.1	mito	nd2	351	84.046	2.80e−87	5,371	5,033
AI939946.1	SAMN00156461	Colon	488	A ↔ T	NC_012920.1	mito	nd2	393	97.710	0.0	5,062	4,670
AI939952.1	SAMN00156802	Colon	263	A ↔ T	NC_012920.1	mito	nd2	236	95.339	8.21e−101	4,958	4,723
AI939954.1	SAMN00156802	Colon	252	A ↔ T	NC_012920.1	mito	nd2	225	96.444	2.95e−100	4,945	4,723
AI939955.1	SAMN00156802	Colon	212	A ↔ T	NC_012920.1	mito	nd2	113	99.115	1.14e−49	4,905	4,794
AI939956.1	SAMN00156802	Colon	200	A ↔ T	NC_012920.1	mito	nd2	186	98.387	6.48e−87	4,930	4,746
AI939957.1	SAMN00156802	Colon	269	A ↔ T	NC_012920.1	mito	nd2	218	97.706	2.28e−101	4,751	4,967
AI939958.1	SAMN00156802	Colon	223	A ↔ T	NC_012920.1	mito	nd2	198	96.465	6.48e−87	4,919	4,723
AI939960.1	SAMN00156802	Colon	286	A ↔ T	NC_012920.1	mito	nd2	245	94.694	2.28e−101	4,962	4,723
AI939964.1	SAMN00156802	Colon	251	A ↔ T	NC_012920.1	mito	nd2	229	96.070	2.95e−100	4,949	4,723
AI939967.1	SAMN00156802	Colon	281	A ↔ T	NC_012920.1	mito	nd2	245	97.551	3.74e−114	4,723	4,967
AI939971.1	SAMN00156802	Colon	251	A ↔ T	NC_012920.1	mito	nd2	240	92.500	1.36e−90	4,962	4,723
AI939974.1	SAMN00156802	Colon	243	A ↔ T	NC_012920.1	mito	nd2	231	94.805	3.74e−96	4,952	4,723
AI939975.1	SAMN00156802	Colon	152	A ↔ T	NC_012920.1	mito	nd2	152	96.711	5.05e−65	4,898	4,748
AI939977.1	SAMN00156802	Colon	226	A ↔ T	NC_012920.1	mito	nd2	201	95.522	1.37e−85	4,922	4,723
AI939978.1	SAMN00156802	Colon	212	A ↔ T	NC_012920.1	mito	nd2	188	97.340	1.77e−84	4,914	4,729
AI939979.1	SAMN00156802	Colon	162	A ↔ T	NC_012920.1	mito	nd2	163	95.706	3.02e−67	4,949	4,789
AI939982.1	SAMN00156802	Colon	248	A ↔ T	NC_012920.1	mito	nd2	233	95.708	3.72e−101	4,955	4,723
AI939984.1	SAMN00156802	Colon	233	A ↔ T	NC_012920.1	mito	nd2	214	94.393	2.93e−87	4,933	4,723
AI939985.1	SAMN00156802	Colon	145	A ↔ T	NC_012920.1	mito	nd2	122	96.721	1.86e−49	4,847	4,967
AI939986.1	SAMN00156802	Colon	166	A ↔ T	NC_012920.1	mito	nd2	163	93.865	8.45e−63	4,891	4,729
AI940581.1	SAMN00156483	Head_neck	533	A ↔ T	NC_012920.1	mito	nd2	392	99.490	0.0	4,671	5,062
AI940582.1	SAMN00156483	Head_neck	509	A ↔ T	NC_012920.1	mito	nd2	393	99.237	0.0	4,670	5,062
AI940585.1	SAMN00156483	Head_neck	475	A ↔ T	NC_012920.1	mito	nd2	394	96.701	0.0	4,670	5,062
AI940586.1	SAMN00156483	Head_neck	296	A ↔ T	NC_012920.1	mito	nd2	294	98.299	5.83e−144	4,692	4,984
AI940587.1	SAMN00156483	Head_neck	474	A ↔ T	NC_012920.1	mito	nd2	393	99.237	0.0	5,062	4,670
BF354521.1	SAMN00160318	Head neck	383	A → T → C → G → A	NC_012920.1	mito	nd4	343	95.335	2.35e−151	11,016	11,354
CK327105.1	SAMN00157633	Colon	474	A ↔ T	NC_012920.1	mito	nd4	391	99.233	0.0	11,611	11,999
BF370011.1	SAMN00160853	Prostate normal	252	C ↔ T	NC_012920.1	mito	nd4	196	96.429	2.65e−85	11,610	11,801
AW062763.1	SAMN00156993	Colon	624	A ↔ T	NM_002568.3	8	poly(A) binding protein cytoplasmic 1 (PABPC1)	387	97.674	0.0	1,882	1,497
AW062774.1	SAMN00156993	Colon	673	A ↔ T	NM_002568.3	8	poly(A) binding protein cytoplasmic 1 (PABPC1)	405	99.753	0.0	1,889	1,485
AW062781.1	SAMN00156993	Colon	197	A ↔ T	NM_002568.3	8	poly(A) binding protein cytoplasmic 1 (PABPC1)	186	99.462	1.36e−90	1,707	1,523
AW062786.1	SAMN00156993	Colon	583	A ↔ T	NM_002568.3	8	poly(A) binding protein cytoplasmic 1 (PABPC1)	405	99.012	0.0	1,889	1,485
AW062790.1	SAMN00156993	Colon	685	A ↔ T	NM_002568.3	8	poly(A) binding protein cytoplasmic 1 (PABPC1)	405	95.309	0.0	1,888	1,485
AW062791.1	SAMN00156993	Colon	533	A ↔ T	NM_002568.3	8	poly(A) binding protein cytoplasmic 1 (PABPC1)	405	99.012	0.0	1,889	1,485
AW062797.1	SAMN00156993	Colon	331	A ↔ T	NM_002568.3	8	poly(A) binding protein cytoplasmic 1 (PABPC1)	321	99.065	2.64e−162	1,485	1,805
AW062798.1	SAMN00156993	Colon	312	A ↔ T	NM_002568.3	8	poly(A) binding protein cytoplasmic 1 (PABPC1)	303	97.690	1.25e−145	1,503	1,805
AW062806.1	SAMN00156993	Colon	472	A ↔ T	NM_002568.3	8	poly(A) binding protein cytoplasmic 1 (PABPC1)	409	99.511	0.0	1,893	1,485
AW062810.1	SAMN00156993	Colon	625	A ↔ T	NM_002568.3	8	poly(A) binding protein cytoplasmic 1 (PABPC1)	625	99.520	0.0	2,430	1,806
AW062811.1	SAMN00156993	Colon	426	A ↔ T	NM_002568.3	8	poly(A) binding protein cytoplasmic 1 (PABPC1)	405	99.753	0.0	1,485	1,889
AW062812.1	SAMN00156993	Colon	420	A ↔ T	NM_002568.3	8	poly(A) binding protein cytoplasmic 1 (PABPC1)	404	99.257	0.0	1,486	1,889
AW062813.1	SAMN00156993	Colon	688	A ↔ T	NM_002568.3	8	poly(A) binding protein cytoplasmic 1 (PABPC1)	405	100.000	0.0	1,889	1,485
AW062814.1	SAMN00156993	Colon	443	A ↔ T	NM_002568.3	8	poly(A) binding protein cytoplasmic 1 (PABPC1)	405	99.012	0.0	1,485	1,889
AW062816.1	SAMN00156993	Colon	536	A ↔ T	NM_002568.3	8	poly(A) binding protein cytoplasmic 1 (PABPC1)	405	99.753	0.0	1,485	1,889
AW062817.1	SAMN00156993	Colon	250	A ↔ T	NM_002568.3	8	poly(A) binding protein cytoplasmic 1 (PABPC1)	221	93.665	4.91e−85	1,574	1,781
AW176937.1	SAMN00156993	Colon	360	A ↔ T	NM_002568.3	8	poly(A) binding protein cytoplasmic 1 (PABPC1)	346	97.977	7.29e−168	1,485	1,825
AW176944.1	SAMN00156993	Colon	603	A ↔ T	NM_002568.3	8	poly(A) binding protein cytoplasmic 1 (PABPC1)	405	99.012	0.0	1,889	1,485
AW176950.1	SAMN00156993	Colon	419	A ↔ T	NM_002568.3	8	poly(A) binding protein cytoplasmic 1 (PABPC1)	86	100.000	3.15e−37	1,804	1,889
AW176951.1	SAMN00156993	Colon	192	A ↔ T	NM_002568.3	8	poly(A) binding protein cytoplasmic 1 (PABPC1)	142	100.000	2.33e−68	1,748	1,889
AW176956.1	SAMN00156993	Colon	348	A ↔ T	NM_002568.3	8	poly(A) binding protein cytoplasmic 1 (PABPC1)	319	99.060	3.41e−161	1,485	1,803
AW176966.1	SAMN00156993	Colon	497	A ↔ T	NM_002568.3	8	poly(A) binding protein cytoplasmic 1 (PABPC1)	405	99.506	0.0	1,889	1,485
AW176967.1	SAMN00156993	Colon	158	A ↔ T	NM_002568.3	8	poly(A) binding protein cytoplasmic 1 (PABPC1)	159	97.484	1.39e−70	1,917	2,074
AW176975.1	SAMN00156993	Colon	481	A ↔ T	NM_002568.3	8	poly(A) binding protein cytoplasmic 1 (PABPC1)	373	97.855	0.0	1,485	1,854
AW176976.1	SAMN00156993	Colon	605	A ↔ T	NM_002568.3	8	poly(A) binding protein cytoplasmic 1 (PABPC1)	354	88.701	2.17e−118	1,834	1,485
AW176977.1	SAMN00156993	Colon	639	A ↔ T	NM_002568.3	8	poly(A) binding protein cytoplasmic 1 (PABPC1)	405	99.259	0.0	1,889	1,485
AW176978.1	SAMN00156993	Colon	434	A ↔ T	NM_002568.3	8	poly(A) binding protein cytoplasmic 1 (PABPC1)	407	99.017	0.0	1,485	1,889
AW176979.1	SAMN00156993	Colon	599	A ↔ T	NM_002568.3	8	poly(A) binding protein cytoplasmic 1 (PABPC1)	406	99.507	0.0	1,485	1,889
AW176980.1	SAMN00156993	Colon	426	A ↔ T	NM_002568.3	8	poly(A) binding protein cytoplasmic 1 (PABPC1)	405	99.012	0.0	1,485	1,889
AW176981.1	SAMN00156993	Colon	599	A ↔ T	NM_002568.3	8	poly(A) binding protein cytoplasmic 1 (PABPC1)	405	99.012	0.0	1,889	1,485
BF333938.1	SAMN00157869	Head_neck	665	A ↔ T	NM_002568.3	8	poly(A) binding protein cytoplasmic 1 (PABPC1)	79	100.000	2.92e−33	1,563	1,485
BF333939.1	SAMN00157869	Head_neck	611	A ↔ T	NM_002568.3	8	poly(A) binding protein cytoplasmic 1 (PABPC1)	319	98.746	1.89e−159	1,803	1,485
BF333940.1	SAMN00157869	Head_neck	509	A ↔ T	NM_002568.3	8	poly(A) binding protein cytoplasmic 1 (PABPC1)	311	91.318	4.33e−116	1,792	1,485

**Table 3:** List of ESTs mapped on non-coding genomic regions and uncharacterized genes.

EST Acc No.	Biosample	Cancer tissue	Length of EST	Swinger transformation	Acc No of aligned sequence	Chromosome	Gene	Aligned Seq. length	%ID	evalue	5'	3'
AI939983.1	SAMN00156802	Colon	379	A ↔ T	NC_000009.12	9	Non-coding region	360	97.778	4.32e−175	120,855,482	1,21E+08
AI939987.1	SAMN00156802	Colon	367	A ↔ T	NC_000009.12	9	Non-coding region	345	97.971	7.29e-168	120,855,464	1,21E+08
AW062766.1	SAMN00156993	Colon	457	A ↔ T	NC_000016.10	16	Non-coding region	313	99.681	9.49e−162	74,318,755	74319067
AW175734.1	SAMN00156993	Colon	328	A ↔ T	NC_000004.12	4	Non-coding region	136	90.441	1.12e−41	131,268,224	1,31E+08
AW175735.1	SAMN00156993	Colon	620	A ↔ T	NC_000004.12	4	Non-coding region	191	95.288	1.78e−79	131,268,167	1,31E+08
AW175739.1	SAMN00156993	Colon	222	A ↔ T	NC_000008.11	8	Non-coding region	139	97.122	1.83e−59	102,600,346	1,03E+08
AW175744.1	SAMN00156993	Colon	506	A ↔ T	NC_000008.11	8	Non-coding region	313	97.764	3.46e−151	88,009,959	88,009,648
AW903506.1	SAMN00159106	Nervous_normal	422	A → T → C → G → A	NC_000006.12	6	Non-coding region	350	97.143	8.28e−166	132,009,130	1,32E+08
AW903535.1	SAMN00159106	Nervous_normal	440	A → T → C → G → A	NC_000013.11	13	Non-coding region	442	99.321	0.0	95,650,104	95,650,545
AW903555.1	SAMN00159106	Nervous_normal	434	A → T → C → G → A	NC_000010.11	10	Non-coding region	436	99.312	0.0	20,554,187	20,553,752
AW903569.1	SAMN00159106	Nervous_normal	430	A → T → C → G → A	NC_000004.12	4	Non-coding region	357	98.599	1.36e−178	17,483,414	17,483,770
AW903572.1	SAMN00159106	Nervous_normal	248	A → T → C → G → A	NC_000013.11	13	Non-coding region	56	100.000	3.66e−20	95,650,461	95,650,406
BE085219.1	SAMN00159564	Breast	314	A → T → C → G → A	NC_000003.12	3	Non-coding region	311	97.749	3.94e−149	169,791,191	1,7E+08
BF332859.1	SAMN00160205	Breast	211	A → T → C → G → A	NC_000020.11	20	Non-coding region	143	90.210	2.12e−42	59,862,273	59,862,140
BF332861.1	SAMN00160205	Breast	364	A → T → C → G → A	NC_000010.11	10	Non-coding region	256	97.266	4.11e−119	8,810,014	8,810,267
BF354533.1	SAMN00160318	Head neck	388	A → T → C → G → A	NC_000005.10	5	Non-coding region	331	99.698	2.29e−171	69,932,968	69,933,298
BF370228.1	SAMN00160862	Prostate_normal	264	A → T → C → G → A	NC_000023.11	x	Non-coding region	195	95.897	2.01e−82	136,928,879	1,37E+08
AW062783.1	SAMN00156993	Colon	640	A ↔ T	NC_000009.12	9	Uncharacterized LOC101927086	557	75.404	5.09e−60	70,473,455	70,472,931
BF798648.1	SAMN00162090	Colon_ins	344	A ↔ T	NC_000003.12	3	Uncharacterized LOC102723512	148	96.622	2.87e−62	4,006,872	4,006,726

### Swinger-Transformed Genes in Different Cancer Tissues

These 347 swinger-transformed RNAs matched 205 known and two uncharacterized genes (**Tables 1**–**3**). These identified swinger RNAs might be artefacts. However, among these 207 genes, swinger RNAs from 10 genes were detected in multiple cancer types and biosamples (**Table 2**). These genes are adenylate kinase 1 (AK1), cystatin B (CSTB), DLG associated protein (DLGAP), heat shock protein family A (Hsp70) member 8 (HSPA8), mediator complex subunits (MED), poly(A) binding protein cytoplasmic 1 (PABPC1), MT-16s rRNA, MT-ND1, MT-ND2 and MT-ND4.

Two A ↔ T-transformed swinger RNAs detected in the colon and head-neck cancer lines mapped on the adenylate kinase 1 (AK1) coding gene (**Table 2**). AK1 plays an important role in tumor suppression and it is often downregulated in cancer cells (Collavin et al., 1999; Janssen et al., 2004; Vasseur et al., 2005; Jan et al., 2019). Three swinger RNAs detected in breast (A → G → C → T → A) and two colon cancer (A ↔ T) lines mapped on cystatin B (CSTB) (**Table 2**). CSTB plays an important role in expression and epigenetic regulation and is downregulated in lung, gastric and colorectal cancers (Zhang et al., 2016; Ma et al., 2017). CSTB promotes cell proliferation, migration and suppresses apoptosis in gastric cancer cells (Zhang et al., 2016). Downregulation of CSTB also promotes gastric cancer (Zhang et al., 2016). Similarly, swinger RNAs for HSPA8 and PABPC1 were identified. We identified two swinger RNAs from the HSPA8 protein-coding region. The carboxy-terminus of Hsc70 interacting protein (CHIP) plays an important role in cancer initiation and progression (Hatakeyama et al., 2005; Kajiro et al., 2009; Gaude et al., 2012) and has an anti-tumor effect in many cancer types including colon and gastric cancers (Kajiro et al., 2009; Ahmed et al., 2012; Wang et al., 2013; Ying et al., 2013; Wang T et al., 2014; Wang Y et al., 2014). Thirty-three swinger RNAs were transcribed from the PABPC1 gene from colon cancer and head-neck cancer biosamples, respectively (**Table 2**). PABPC proteins are RNA processing proteins associated with gene expression regulation (Liu et al., 2012) and are upregulated in prostate and colorectal cancers (Eisermann et al., 2015). PABPC also has a tumor suppressor role in head and neck squamous cell carcinoma (Zeng et al., 2018). Swinger-transformed RNAs produced from these identified genes will produce non-homologous and putatively nonfunctional mRNAs translating dysfunctional nonhomologous proteins, which supports the association between downregulation of these genes and cancer cell types. These swinger RNAs could be early stage factors responsible for cancer induction or result from genetic instability in later stages of malignant cancers (Ruckova et al., 2012; Ciocca and Calderwood, 2005). Results favor the former because the swinger transformed mRNA of tumor suppressor genes would result in disruption of gene function inhibiting programmed cell death. Analyses also identify two swinger RNAs mapped on DLG associated proteins (DLGAP1 and DLGAP4) coding genes in healthy nervous tissue samples and colon cancer cells. DLGAP is a protein overexpressed in the brain (Fagerberg et al., 2014) and promotes invasiveness in cancer cell lines (Li et al., 2018). Similarly, three swinger RNAs were transcribed from mediator complex subunit genes (MED) in colon and breast cancer tissues (**Table 2**). Mutations in MED12 are associated with tumorigenesis (Bullerdiek and Rommel, 2018; Xie et al., 2018) and cause benign breast fibroepithelial lesions (Pareja et al., 2019). These two observations suggest that swinger transformation of MED should induce carcinogenesis, whereas, swinger RNAs are transcribed from overexpressing DLGAP genes due to enzyme fatigue in cancer cells.

Among the detected swinger RNAs, 51 swinger RNAs match mitochondrial genes (**Tables 1** and **2**). It is widely known and proven that mitochondrial genes are overexpressed in various cancer types to meet up the metabolic requirements of cancer cells and are strongly associated with cancer (Tarantul et al., 2001; Modica-Napolitano and Singh, 2004; Księżakowska-Łakoma et al., 2014; Lin et al., 2018). Among 51 swinger RNAs, 7, 1, 30 and 3 swinger RNAs are transcribed from mitochondrial 16s rRNA, ND1, ND2 and ND4, respectively. These were identified in several biosamples (**Table 2**). Sixty-six percent (33 ESTs) of the identified swinger RNAs were transcribed from the MT-ND region. A previous study showed bias for swinger transformation of MT-ND genes (Warthi and Seligmann, 2018).

Interestingly, detection of swinger transformed DLGAP1 mRNAs (overexpressed ~15× in neurons compared to other tissue cells; Fagerberg et al., 2014), in normal nervous tissue samples suggests that the swinger transformation is not directly associated with cancer but perhaps associated with highly expressed genes. Carcinogenesis could be the outcome of swinger transformed dysfunctional mRNAs. Previously identified swinger RNAs from highly active cancer-associated genes (Warthi et al., 2019) also support this finding. Indeed, even within mitochondrial genes, rRNAs are more expressed than other genes, and swinger rRNAs are the most frequently observed mitochondrial swinger RNAs in previous publications (Seligmann, 2013b; Seligmann, 2013c; Seligmann, 2014b; Seligmann, 2015b), matching the pattern of positive association between regular and swinger transcriptions. Hence, the positive association between expression and swinger transformation occurs independently for human nuclear and mitochondrial genes. Hence, at least at this qualitative level of analysis, observations on these highly expressed nuclear and mitochondrial genes support that swinger transformations associate with highly active genomic regions that result in cancer induction and progression due to nonfunctional transcripts.

In order to test whether swinger transformations are biased towards some regions of genes, genes for which swinger RNAs were detected in more than one biosample (**Table 2**) are regionally compartmentalized in three equal regions i.e. 5' region, mid region and 3' regions, each region spanning 1/3 of the gene. Mitochondrial genes like 16S rRNA (five of seven ESTs), ND4 (two of three ESTs), and nuclear gene Cystatin B (three of three ESTs) mapped on the 3' extremity of their respective genes.

Among the 30 ESTs mapped on ND2 gene across multiple biosamples, 28 mapped on the central region (5' to 3'; 4,692 to 4,984 bp) of the mitochondrial genome (NC_012920.1), while two mapped on the 5' terminal of ND2 (4,482–4723 bp) gene. Interestingly, for MT-ND4, among the three ESTs identified across three different biosamples for three different swinger transformations, two ESTs mapped on the same 3' terminal ND4 region i.e. 11,610–11,801 bp on the mitochondrial genome (NC_012920.1). For Nuclear gene poly(A) binding protein cytoplasmic 1 (PABPC1), all the 33 identified ESTs preferentially mapped on the mid-region (5' 1,485 bp to 3' 1,803 bp on NM_002568.3) of PABPC1 gene.

Considering mitochondrial and nuclear genes separately, 38 among 43 (mitochondrial, 88.4%, one tailed sign test P = 0.0011) and 33 among 33 (nuclear, 100%, one tailed sign test P = 1.6 × 10^−6^) swinger RNAs map either on the mid or the 3' regions of the gene. These tests assume that the probability of mapping randomly on these regions is 2/3. One tailed sign tests are justified by the working hypothesis that polymerase enzyme fatigue (occurring more downstream from transcription initiation) causes swinger transcription. These observations indicate that the mid and 3' regions of highly expressed genes are more prone to produce swinger transformed RNAs than the 5' region, for each nuclear and mitochondrial genes. Similarly, swinger transformed ESTs (from only one biosample) mapping on identified genes, preferentially mapped on the same region (**Table 1** shows the 5' and 3' positions of such ESTs).

Among 275 ESTs from nuclear protein coding genes, 196 ESTs mapped on exons of genes and 76 swinger transformed ESTs mapped on gene introns. Three ESTs mapped partly on gene exons and partly on introns, suggesting that swinger transformations occur before post transcriptional modification, and support our working hypothesis of swinger polymerization during replication/transcription.

The 347 ESTs correspond to 203 protein coding genes and 19 RNAs from non-coding regions. The median and mean size of a protein coding gene in human genome are ~26,288 bp and ~66,577 bp (Piovesan et al., 2016). The mean and median length of identified 203 swinger transformed protein coding genes (including mitochondrial genes) are 124,427 bp and 55,291 bp which is almost twice the mean and median lengths of human protein coding genes. These genes for which swinger transcripts were detected also included the largest protein coding gene in the human genome i.e. RNA binding fox-1 homolog 1 (RBFOX1). These observations indicate that swinger transformation probably due to polymerase fatigue is not only associated to highly active genes but could also associate with large genes. This could also explain the biased mapping of swinger RNAs on the mid and 3' regions of genes, vs the first third of genes.

### Swinger RNAs From Non-Coding and Uncharacterized Genomic Regions

Seventeen and two identified swinger RNAs were transcribed from 15 non-coding genomic regions and two uncharacterized genomic regions, respectively (**Table 3**). The 15 non-coding swinger RNAs were not from 5' or 3' UTR or intronic regions of protein-coding genes. To test if these 15 identified genomic regions on various human chromosomes are transcriptionally active, we did a transcriptome search in seventy-one samples (SRX768406–SRX768476, Garzon et al., 2014) with the identified swinger RNAs transcribed from these non-coding genomic regions. Good and complete alignments of the reads on the searched sequences confirmed transcriptions of these 15 non-coding regions (**Supplementary Table 2**). This result suggests that these 15 regions are transcriptionally active, with potential roles in nerve cells and possible associations with carcinogenesis. However, these specific cancer tissue samples are unavailable, preventing further *in-vitro* and *in-vivo* tests and analyses.

The identified swinger RNAs were mapped on human nuclear chromosomes. No compartmentalizations on any chromosomal arm, region or band of a chromosome were detected in relation to swinger expressed regions. Swinger RNAs produced by transformations that are more conservative at the amino acid level are also more abundant than swinger RNAs produced by transformations that cause more amino acid changes (Seligmann, 2018). This effect was also observed when comparing transformations involving the same number of nucleotides in the transformation (2, 3 or 4). For the distribution of swinger RNAs detected in the present study, too few classes of transformations were detected to enable such subdivisions. However, when considering all transformations, conservation increases with swinger RNA abundances (r = 0.4187, one tailed P = 0.0234). C ↔ G is the least conservative among the transformations involving only two nucleotides, and is one among two transformations for which no swinger RNA was detected in the currently examined data.

## Conclusion

We report genomic origins of 347 previously unidentified ESTs generated by the FAPESP/LICR Human Cancer Genome Project (Neto et al., 2000). These represent 0.23% of the 149,500 unidentified ESTs from that project. Note that other types of systematic transformations apparently produce non-canonical RNAs, such as systematic deletions, which produce so-called delRNAs (Seligmann, 2015c; Seligmann, 2016f; El Houmami and Seligmann, 2017; Seligmann, 2017b; Warthi and Seligmann, 2019) and corresponding peptides (Seligmann, 2015c; Seligmann, 2016a; Seligmann, 2016b; Seligmann, 2016c; Seligmann, 2016d; Seligmann, 2016e), including chimeric peptides (Seligmann and Warthi, 2019). This underlines a general approach to identify unknown sequences generated by various sequencing methods. This study identifies swinger RNAs transcribed from multiple cancer-associated genes (**Tables 1** and **2**), suggesting highly active genes produce swinger transcripts possibly due to enzyme fatigue and promoting cancer progression. The identified sequences might be sequencing artefacts. However, random artefacts generated by sequencing equipment could not produce systematic exchanges. Systematic sequencing errors should produce swinger RNAs for all sequenced ESTs, however, biosamples where swinger RNAs are detected include regular canonical RNAs. Analyses also identify transcriptionally active non-coding regions on human chromosomes discovering putative ncRNA transcribing regions with potentially significant roles in normal and cancer cells. We provide a unique method to study and identify unknown sequencing reads, reducing loss of important genetic information in raw sequence data. Systematic editing of RNA might contribute to solve the dark DNA conundrum.

## Data Availability Statement

All datasets generated for this study are included in the article/**Supplementary Material**.

## Author Contributions

GW conducted design, analysis, observation and writing. P-EF contributed to result assessments. HS designed the systematic nucleotide exchange framework. P-EF and HS contributed to writing and supervising this study.

## Funding

This work was supported by the A*MIDEX project (no ANR-11-IDEX-0001-02) funded by the « Investissements d'Avenir » French Government program, managed by the French National Research Agency (ANR).

## Conflict of Interest

The authors declare that the research was conducted in the absence of any commercial or financial relationships that could be construed as a potential conflict of interest.

## Supplementary Material

Supplementary Table 1 Swinger transformed version of 347 ESTs mapped on human genome.

Supplementary Table 2 Whole cell transcriptome BLAST result for swinger transformed ESTs mapped on non coding genomic region.
